# The Effectiveness of Topical Treatment for Plantar Warts: A Retrospective Cohort Study

**DOI:** 10.3390/idr16060090

**Published:** 2024-11-26

**Authors:** Ana Mª Rayo Pérez, José María Juárez Jiménez, Rafael Rayo Rosado, Raquel García de la Peña

**Affiliations:** Department of Podiatry of the Faculty of Nursing, Physiotherapy and Podiatry of the University of Seville, Calle Avicena s/n, 41009 Seville, Spain; jmjuarez@us.es (J.M.J.J.); rafaelrayo@us.es (R.R.R.); raquelgp@us.es (R.G.d.l.P.)

**Keywords:** HPV, plantar wart, treatment, effectiveness, nitric acid, cantharidin, bleomycin

## Abstract

**Background**: Plantar warts, caused by human papillomavirus (HPV), are a common condition that can be painful and resistant to treatment. There are various therapeutic options for managing them, but it is not always clear which are the most effective and tolerated by patients. Among the most commonly used treatments are a zinc and nitric complex (nitrizinc complex), cantharidin, and bleomycin, each with different mechanisms of action and profiles in terms of pain and patient satisfaction. **Objectives**: We aimed to evaluate and compare the clinical efficacy, post-treatment pain, and patient satisfaction among three common treatments (zinc and nitric complex, cantharidin, and bleomycin) in subjects with plantar warts, as well as identify the most effective and best-tolerated treatment. **Materials and Methods**: This is a retrospective case series study analyzing 60 records of subjects aged 18 to 40 years diagnosed with plantar warts without systemic diseases or allergies and without any prior treatment. Complete records from 2020 to 2023 were selected. Subjects were divided into three groups according to the treatment received (zinc and nitric complex, cantharidin, bleomycin), and demographic variables, post-treatment pain (measured using the visual analog scale), the number of sessions required, and satisfaction after discharge (evaluated with the Likert scale) were analyzed. **Results**: Of the 60 subjects included, the group treated with bleomycin experienced higher levels of pain after the first session (mean of 7.1 points on the VAS) compared to the cantharidin group (2.7 points) and the zinc and nitric complex group (1.1 points). However, the bleomycin group required fewer sessions for complete healing (an average of 1.8 sessions), while the nitric acid group needed more (3.4 sessions), with cantharidin falling in between (2.5 sessions). Regarding post-discharge satisfaction, all groups showed comparable scores (between 7.9 and 8.5 points), although cantharidin demonstrated slightly higher satisfaction. A statistical analysis showed significant differences in the number of sessions and post-treatment pain between treatments (*p* < 0.05) but not in final satisfaction. **Conclusions**: Although bleomycin treatment is more painful, it is the most effective in terms of reducing the number of sessions required for complete healing. Cantharidin offers a good balance between efficacy and patient satisfaction, while a zinc and nitric complex, although less painful, requires more sessions for complete treatment. Each treatment has specific advantages, suggesting that therapeutic choices should be personalized according to the patient’s needs and preferences.

## 1. Introduction

Plantar warts are benign hyperplastic epidermal lesions caused by human papillomavirus (HPV). These types of lesions affect approximately 10–12% of the population, with 6% being plantar warts. These viruses are epidermotropic, meaning they do not spread systemically [1,2].

Infection occurs through direct contact with the virus, either via contaminated surfaces or infected skin, and is particularly favored by warm and humid environments. As such, the main risk factors include walking barefoot in public places, poor hygiene, hyperhidrosis, swimming in heated pools, a compromised immune system, or tropical climates [3].

Plantar warts typically appear as circular, flat, annd papular lesions measuring 0.5–5 mm in diameter and are usually skin-colored or brownish. They are generally asymptomatic and tend to develop in weight-bearing areas. They may present as single lesions or as multiple lesions [1,4,5].

**Figure 1:**
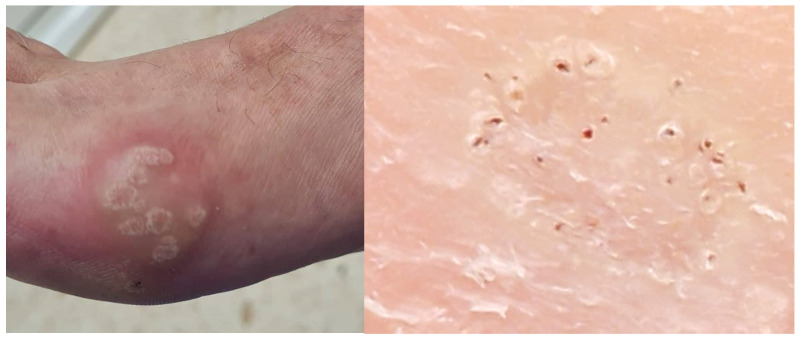
Clinical image and dermatoscopy of a plantar wart. Source: own.

Diagnosis is clinical, using the pinch test, loss of dermatoglyphic patterns, and pinpoint bleeding (Figure 1). The diagnosis can be confirmed through pathological anatomy, dermatoscopy, or even ultrasound [6].

Among the available treatments, there are multiple therapeutic options depending on the method of application. Regarding physical treatments, the most commonly used are cryotherapy, with an effectiveness rate of 50–70%, and laser therapy, with an effectiveness rate of 60–70%. As for immunomodulatory treatments, Falknor’s multipuncture technique has an effectiveness rate of 75–85%. Despite the high effectiveness rates of these treatments, pharmacological treatments are the preferred choice for plantar warts. In cases where all the aforementioned treatments fail, surgical treatment should be established through curettage or complete excision of the lesion, but these procedures can be the origin of hypertrophic scars if proper skin management is not carried out. It is important to note that more aggressive treatments tend to have higher effectiveness rates but also cause greater pain during the process. Therefore, treatment should be tailored based on the characteristics of each individual [7,8,9].

**Figure 2:**
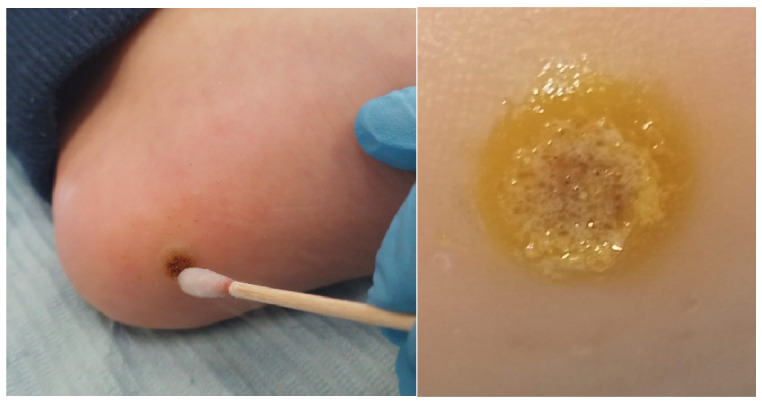
Application of nitric acid. Source: Own.

Focusing on chemical treatments, the most commonly used is a zinc and nitric complex (Figure 2). This chemical agent destroys the virus by coagulating the tissue. Its application can cause skin irritation and burns. It has an effectiveness rate of 65–85%. The application protocol consists of serial applications of this complex using swabs for 30–60 s on the lesion until a yellowish halo forms. Applications are performed every 7–10 days until resolution of the condition [10,11].

**Figure 3:**
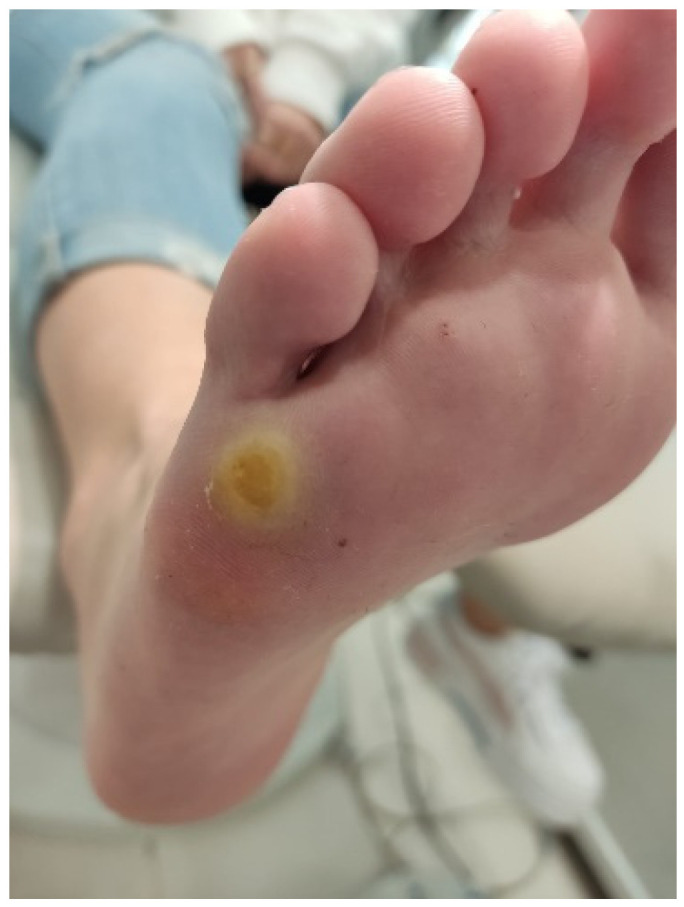
Application of cantharidin. Source: own.

Cantharidin is a natural vesicant compound prepared as a magistral formulation and consists of a combination of 1% cantharidin, 30% salicylic acid, 5% podophyllin, and flexible collodion q.s. 2 mL. It is applied topically, causing blister formation that helps eliminate the virus (Figure 3). Although this treatment can cause discomfort, it is generally well tolerated by patients and has an effectiveness rate of 80–100%. It is applied directly to the lesion and after 20–30 s, when the collodion dries, the area is covered with a porous dressing. After 48 h, the blister is removed and serial dressing changes are performed until complete skin healing. If the lesion persists, another application is performed after 20–30 days [12,13,14].

**Figure 4:**
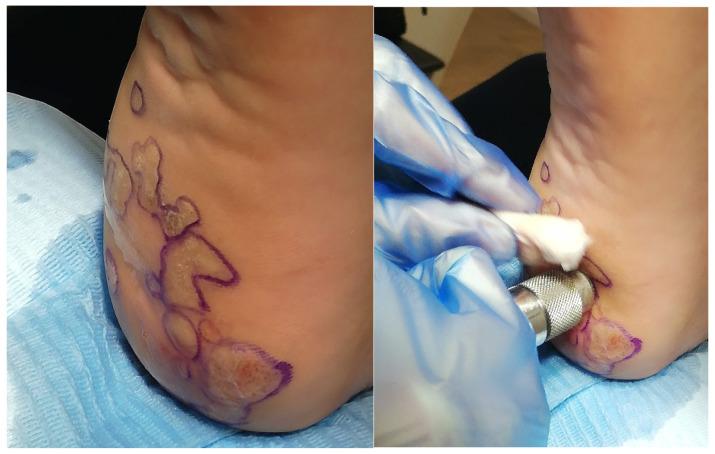
Application of bleomycin. Source: own.

Finally, bleomycin is discussed. This chemotherapeutic agent is administered through intralesional injections (Figure 4). It works by inhibiting DNA synthesis in HPV-infected cells. While highly effective, it is associated with certain adverse effects such as local necrosis and scarring. For its application, a dermojet syringe is used, which delivers the drug into the epidermis using pressurized air. Multiple applications are made over the entire surface of the lesion, so it is recommended to perform the procedure under local anesthesia. A follow-up is conducted after 7–10 days, and if the lesion persists, another application is performed. It has an effectiveness rate of 80–90%, although post-procedure pain is around 8–10 points on the visual analog scale [15,16].

There are numerous clinical challenges associated with the treatment of plantar warts. The high recurrence rate and variability in treatment response make it difficult to find a single therapeutic strategy that works for every individual, often presenting a significant problem in clinical practice. Additionally, the pain and complications caused by many available treatments can deter patients from seeking medical attention, which may prolong symptoms and contribute to the spread of the virus [17,18].

To guide clinical practice and improve treatment outcomes, this study evaluates and compares the clinical efficacy, post-treatment pain, and patient satisfaction among three common treatments (zinc and nitric complex, cantharidin, and bleomycin) in subjects with plantar warts, aiming to identify the most effective and best-tolerated treatment.

## 2. Materials and Methods

### 2.1. Study Design

A retrospective observational cohort study was conducted, reviewing the medical records of subjects who visited podiatry consultations for plantar warts between 2020 and 2023. The purpose of the study was to evaluate the effectiveness and pain associated with different treatments applied to these types of lesions. The treatments were divided into three groups, namely those treated with a zinc and nitric complex (Group A), cantharidin (Group B), and bleomycin (Group C).

### 2.2. Inclusion and Exclusion Criteria

Several inclusion criteria were established for the selection of subjects in the study. Participants had to be between 18 and 40 years old and have a confirmed diagnosis (clinical signs and ultrasound) of plantar warts. Additionally, they had to be free from systemic diseases, allergies, or a history of COVID-19, and they must not have received any prior treatment in the affected area. The availability of complete clinical records documenting the progression of the lesion over time was also required.

On the other hand, subjects with incomplete clinical records or those that lacked the necessary information for proper treatment follow-up were excluded from the study. Individuals with a history of previous treatments in the affected area without clear documentation were also excluded, as were those who had received multiple treatments without a detailed follow-up of each intervention performed.

### 2.3. Study Variables

The main variables analyzed in the study were as follows:

(1) Treatment efficacy, measured as the time required for complete healing of the lesion.
(2) Post-treatment pain after the first session, assessed using the visual analog scale (VAS) for pain, with a score ranging from 0 to 10.
(3) Number of treatment sessions required for the complete resolution of the plantar wart.
(4) Type of treatment, namely a zinc and nitric complex, cantharidin, or bleomycin.
(5) Location of the plantar wart, documented based on its anatomical position.
(6) Demographic data, namely age, body mass index (BMI), and sex.
(7) Plantar wart size, measured using an automatic caliper.

### 2.4. Data Collection

Clinical information was obtained from medical records between 2020 and 2023. Only subjects whose records met the inclusion criteria were included. The subjects were grouped into three categories according to the treatment received as follows:

Group A: subjects treated with a zinc and nitric complex (*n* = 20).

Group B: subjects treated with cantharidin (*n* = 20).

Group C: subjects treated with bleomycin (*n* = 20).

### 2.5. Procedure

(1) Record selection: A screening of medical records was conducted to identify those that met the established criteria. Subsequently, subjects were assigned to the corresponding groups based on the treatment received.
(2) Data extraction: data regarding the previously defined variables (pain, number of sessions, lesion size, satisfaction, etc.) were extracted.
(3) Comparative analysis: once the data were grouped by treatment, the following key variables were compared:

Healing rate: the effectiveness of each treatment was evaluated by the number of sessions required for the complete resolution of the lesion.

Post-treatment pain: pain levels reported by subjects after the first session of each treatment were compared.

Subject satisfaction: assessed using the Likert scale, used as an indirect parameter of the perceived effectiveness by the subjects.

### 2.6. Statistical Analysis

Data analysis was performed using Jamovi software (version 2.26.0, The jamovi Project, Australia). The following analyses were carried out:

Means, medians, standard deviations, and ranges were calculated for demographic and clinical variables, including age, BMI, lesion size, number of sessions, post-treatment pain, and satisfaction.

To compare efficacy and post-treatment pain between the different treatment groups, an analysis of variance (ANOVA) was performed. This test was used to assess whether there were significant differences in post-treatment satisfaction, the number of sessions required, and pain among the three groups.

The Tukey test was applied for multiple group comparisons to identify specific differences between treatments in terms of post-treatment satisfaction and pain. A *p*-value of <0.05 was considered significant. The F-value and *p*-value were used to evaluate the presence of significant differences between groups concerning key variables.

### 2.7. Ethical Considerations

The study was conducted following ethical guidelines for research involving humans, ensuring the confidentiality of subjects’ clinical data. All subjects included in the study had signed informed consent for the treatment and monitoring of their lesions. This study did not involve new interventions on the subjects, as it only used previously obtained records.

## 3. Results

The study included a total of 60 subjects divided into three groups with similar demographic characteristics. The mean age of the total sample was 26.6 years (SD ± 6.0), with an equitable distribution by sex. The average body mass index (BMI) was 23.4 kg/m^2^ (SD ± 0.9).

Regarding the warty lesions, the average size was 5.1 mm (SD ± 1.2). Subjects required an average of 2.5 treatment sessions (SD ± 1.1), and the intensity of pain after the first session was 4.8 points (SD ± 2.4) on the visual analog scale (VAS). Subject satisfaction at the end of the treatment was 8.3 points (SD ± 1.1) on the Likert scale. The following Table 1 presents a descriptive analysis of the total sample:

### 3.1. Comparison Between Treatment Groups

Regarding post-treatment pain (Table 2), subjects treated with bleomycin reported higher pain levels after the first session, with a mean of 7.1 compared to 2.7 in the cantharidin group and 1.1 in the zinc and nitric complex group. Despite this, post-treatment satisfaction (Tabla 2) was similar across all groups, with means ranging from 7.9 to 8.5 points. The number of sessions (Table 2) required to complete treatment varied by group, being higher in the zinc and nitric complex group (3.4) compared to bleomycin (1.8) and cantharidin (2.5).

### 3.2. Statistical Analysis

**Figure 5:**
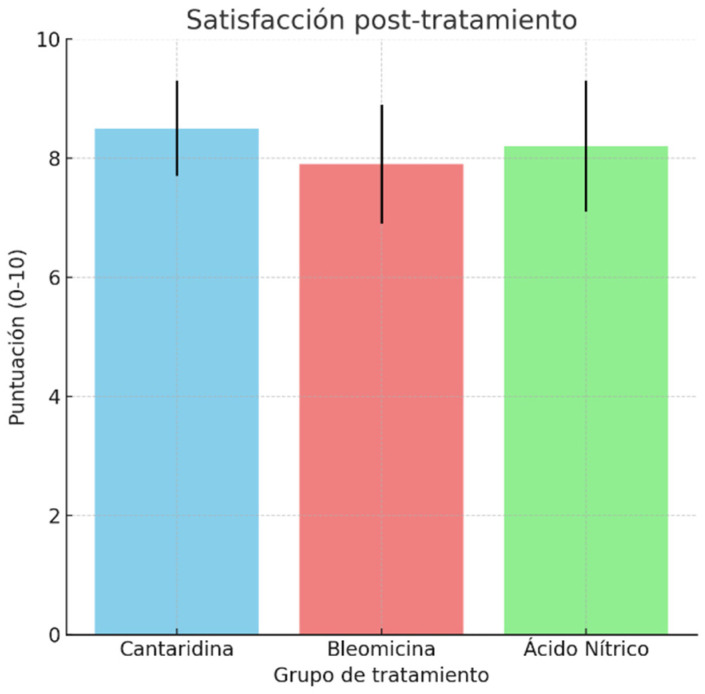
Post-treatment satisfaction.

Satisfaction after discharge: the ANOVA did not reveal significant differences in satisfaction among the three treatment groups (F = 2.86, *p* = 0.066), indicating comparable effectiveness in terms of satisfaction across the treatments (Figure 5).

Number of sessions: There were significant differences in the number of sessions among the groups (F = 10.56, *p* < 0.001). Subjects treated with bleomycin required significantly fewer sessions than those treated with a zinc and nitric complex, with cantharidin in an intermediate position.

**Figure 6:**
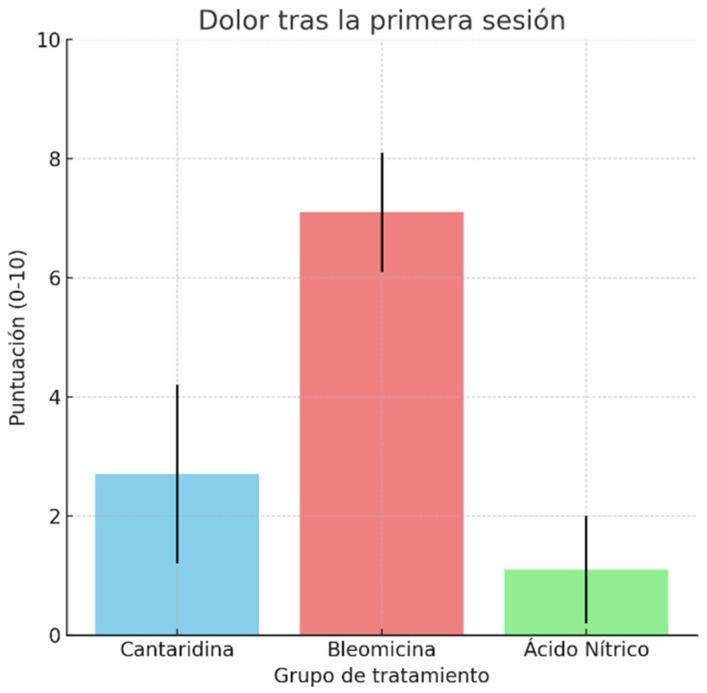
Pain after the first session.

Reported pain: The ANOVA showed significant differences in reported pain among the groups (F = 125.67, *p* = 0.001). Subjects treated with bleomycin experienced significantly more pain compared to those treated with cantharidin and a zinc and nitric complex, with the latter being the least painful treatment (Figure 6).

### 3.3. Tukey Test

The Tukey multiple comparisons test revealed that satisfaction was significantly higher in the cantharidin group compared to the bleomycin group (*p* = 0.048). No significant differences were found between cantharidin and the zinc and nitric complex, nor between bleomycin and the zinc and nitric complex (Table 3).

## 4. Discussion

This study conducted a comparison of three pharmacological treatments employed in the management of plantar warts, namely nitric acid, bleomycin, and cantharidin, with the aim of providing a comprehensive assessment of their effectiveness, associated pain, and complications. The results reveal variations between treatment with nitric acid, the cantharidin master formula, and bleomycin in terms of the number of sessions for healing, post-treatment pain, and patient satisfaction, underscoring the need for an individualized approach to treatment selection.

The zinc and nitric complex has demonstrated efficacy in the treatment of plantar warts, with reported success rates around 90% according to Janniger et al. (2017) [19]. This treatment is distinguished by being less aggressive than alternative options, with minimal adverse effects. However, one of the challenges in its application is the necessity for repeated sessions over extended periods, which could affect subjects’ adherence, particularly in those with low pain tolerance or impatience for quick results. Additionally, Cusini et al. (2015) [20] noted that the effectiveness of nitric acid was significantly higher for individual warts than for multiple lesions, suggesting that the number and size of the lesions may influence outcomes. Therefore, its use may be more suited for subjects with isolated lesions or for those who prefer a less invasive option at the expense of a more prolonged treatment.

Despite the lower rate of adverse effects, the primary limitation of nitric acid is the prolonged duration of treatment, which may impact the patient’s experience. Studies have reported no significant adverse effects, making it a viable option for pediatric patients or those with low pain tolerance. However, in the context of adult patients who are more urgently seeking to resolve the lesion, faster treatments such as cantharidin or bleomycin may be more appropriate [18,19,20].

Cantharidin has been extensively used due to its rapid action and ease of application. In our study, we found an effectiveness exceeding 80%, which aligns with the results reported by Vakharia et al. (2018) [21]. However, a high incidence of adverse effects was observed, including significant pain and changes in skin pigmentation (hyperpigmentation or hypopigmentation), affecting up to 53% of treated cases. Keam et al. (2024) [22] also confirmed that 0.7% cantharidin is highly effective not only for plantar warts but also for molluscum contagiosum, thereby expanding its applicability to other viral skin infections.

Nevertheless, an important factor to consider is that the speed of healing associated with cantharidin comes with an increase in post-treatment discomfort. The pain associated with blister formation and the necrosis of lesions can be significant for some patients, which may limit its use in sensitive populations such as children or individuals with low pain thresholds. However, for those patients seeking rapid results and willing to tolerate a temporary level of pain, cantharidin remains a valuable option [22].

It is also important to note that although the rate of adverse effects is high, most are temporary and do not result in long-term complications. This, along with the possibility of achieving complete resolution in just a few sessions, makes cantharidin a preferred option for those patients who prioritize the speed of healing [21,22].

Bleomycin, used both intralesionally and in combination with electroporation, has shown to be an effective option in the management of plantar warts, with a success rate of 95% in some studies. Pasquiali et al. (2017) [23] noted that the combination with electroporation significantly increases the drug’s penetration into the lesions, which may explain its greater effectiveness compared to bleomycin alone. However, Di Chiacchio et al. (2019) [24] observed that electroporation can be associated with an increase in adverse effects, such as pain during and after application, without resulting in a significant improvement in effectiveness in all cases.

The main drawback of bleomycin is the intensity of the pain during injection and in the days following treatment, which can be a limiting factor for some patients. However, the benefit of bleomycin is its ability to eliminate warts in fewer sessions than nitric acid, and with a lower incidence of persistent adverse effects compared to cantharidin. This makes it especially useful for patients with large or treatment-resistant warts, provided they can tolerate the associated pain of its administration [22,25].

Moreover, recent studies suggest that the effectiveness of bleomycin may largely depend on the type of wart and its location. Plantar warts tend to be more resistant to conventional treatments, and bleomycin offers a potent alternative when other methods fail. However, it should be carefully considered in patients with low pain tolerance or those requiring a less invasive approach [21,22].

The selection of the ideal treatment for plantar warts should take into account several factors, such as effectiveness, treatment duration, pain level, and possible complications. In terms of effectiveness, both cantharidin and bleomycin have been demonstrated to be faster and more efficient than nitric acid but at the cost of greater discomfort and transient side effects. Nitric acid, on the other hand, offers a more conservative option with fewer adverse effects, although it requires a greater commitment in terms of treatment duration [24,25,26].

It is important to emphasize that although numerous studies have been conducted on these treatments, there remains a lack of direct comparisons that consistently evaluate both effectiveness and pain tolerance, as well as side effects in a diverse population of subjects. This highlights the need for more large-scale and high-quality research that can provide stronger evidence-based recommendations.

## 5. Conclusions

The results obtained show that both cantharidin and bleomycin provide a quicker cure compared to nitric acid, but at the expense of greater pain and the occurrence of adverse effects. Although the differences in perceived effectiveness between the treatments are not significant, the patient’s profile and pain tolerance play a crucial role in choosing the most appropriate treatment. Thus, the selection of treatment should be based on a balance between effectiveness, treatment duration, and patient tolerance.

## Figures and Tables

**Table 1:** Descriptive analysis of the total sample.

Variable	Mean	Median	SD	Range
Age (years)	26.6	27	6.0	18–40
BMI (kg/m^2^)	23.4	23.5	0.9	21.0–25.2
Lesion size (mm)	5.1	5	1.2	2–7
Number of sessions	2.5	2	1.1	1–5
Pain after 1st session (0–10)	4.8	5	2.4	0–9
Satisfaction after discharge (0–10)	8.3	8	1.1	6–10

**Table 2:** Comparison among three treatment groups.

Variable	Total Sample	Cantharidin	Bleomycin	Zinc and Nitric Complex
Age (years)	25.5	26.9	26.0	25.9
BMI (kg/m^2^)	23.3	23.4	23.4	23.3
Lesion Size (mm)	5.2	4.9	5.3	5.1
Number of Sessions	2.5	2.5	1.8	3.4
Pain after 1st Session (0–10)	2.7	7.1	1.1	4.0
Satisfaction after Discharge (0–10)	8.5	7.9	8.2	8.1

**Table 3:** Results of the Tukey test for post-treatment satisfaction.

Comparison	*p*-Value
Cantharidin vs. Bleomycin	*p* = 0.048
Cantharidin vs. Zinc and Nitric Complex	*p* = 0.521
Bleomycin vs. Zinc and Nitric Complex	*p* = 0.137

## Data Availability

Not report any data for this study.
